# Features of immunometabolic depression as predictors of antidepressant treatment outcomes: pooled analysis of four clinical trials

**DOI:** 10.1192/bjp.2023.148

**Published:** 2024-03

**Authors:** Sarah R. Vreijling, Cherise R. Chin Fatt, Leanne M. Williams, Alan F. Schatzberg, Tim Usherwood, Charles B. Nemeroff, A. John Rush, Rudolf Uher, Katherine J. Aitchison, Ole Köhler-Forsberg, Marcella Rietschel, Madhukar H. Trivedi, Manish K. Jha, Brenda W. J. H. Penninx, Aartjan T. F. Beekman, Rick Jansen, Femke Lamers

**Affiliations:** Department of Psychiatry, Amsterdam University Medical Center, Vrije Universiteit Amsterdam, Amsterdam, The Netherlands; and Mental Health Program, Amsterdam Public Health, Amsterdam, The Netherlands; Department of Psychiatry, University of Texas Southwestern Medical Center, Dallas, Texas, USA; Department of Psychiatry and Behavioral Sciences, Stanford School of Medicine, Stanford University, Stanford, California, USA; Department of General Practice, Westmead Clinical School, University of Sydney, Sydney, Australia; Westmead Applied Research Centre, Faculty of Medicine and Health, University of Sydney, Sydney, Australia; and George Institute for Global Health, Sydney, Australia; Department of Psychiatry and Behavioral Sciences, Dell Medical School, University of Texas, Austin, Texas, USA; Department of Psychiatry and Behavioral Health, Duke School of Medicine, Durham, North Carolina, USA; and Duke-National University of Singapore, Singapore, Singapore; Department of Psychiatry, Dalhousie University, Halifax, Nova Scotia, Canada; Departments of Psychiatry & Medical Genetics, College of Health Sciences, University of Alberta, Edmonton, Alberta, Canada; Neuroscience and Mental Health Institute, University of Alberta, Edmonton, Alberta, Canada; and Women and Children's Research Institute, University of Alberta, Edmonton, Alberta, Canada; Psychosis Research Unit, Aarhus University Hospital Psychiatry, Aarhus, Denmark; and Department of Clinical Medicine, Aarhus University, Aarhus, Denmark; Department of Genetic Epidemiology in Psychiatry, Central Institute of Mental Health, Faculty of Medicine Mannheim, University of Heidelberg, Mannheim, Germany; Department of Psychiatry, Amsterdam University Medical Center, Vrije Universiteit Amsterdam, Amsterdam, The Netherlands; Mental Health Program, Amsterdam Public Health, Amsterdam, The Netherlands; and Mood, Anxiety, Psychosis, Sleep & Stress Program, Amsterdam Neuroscience, Amsterdam, The Netherlands; Department of Psychiatry, Amsterdam University Medical Center, Vrije Universiteit Amsterdam, Amsterdam, The Netherlands; and Mood, Anxiety, Psychosis, Sleep & Stress Program, Amsterdam Neuroscience, Amsterdam, The Netherlands

**Keywords:** Antidepressants, depressive disorders, treatment, profiling, inflammation

## Abstract

**Background:**

Profiling patients on a proposed ‘immunometabolic depression’ (IMD) dimension, described as a cluster of atypical depressive symptoms related to energy regulation and immunometabolic dysregulations, may optimise personalised treatment.

**Aims:**

To test the hypothesis that baseline IMD features predict poorer treatment outcomes with antidepressants.

**Method:**

Data on 2551 individuals with depression across the iSPOT-D (*n* = 967), CO-MED (*n* = 665), GENDEP (*n* = 773) and EMBARC (*n* = 146) clinical trials were used. Predictors included baseline severity of atypical energy-related symptoms (AES), body mass index (BMI) and C-reactive protein levels (CRP, three trials only) separately and aggregated into an IMD index. Mixed models on the primary outcome (change in depressive symptom severity) and logistic regressions on secondary outcomes (response and remission) were conducted for the individual trial data-sets and pooled using random-effects meta-analyses.

**Results:**

Although AES severity and BMI did not predict changes in depressive symptom severity, higher baseline CRP predicted smaller reductions in depressive symptoms (*n* = 376, β_pooled_ = 0.06, *P =* 0.049, 95% CI 0.0001–0.12, *I*^2^ = 3.61%); this was also found for an IMD index combining these features (*n* = 372, β_pooled_ = 0.12, s.e. = 0.12, *P =* 0.031, 95% CI 0.01–0.22, *I*^2^ *=* 23.91%), with a higher – but still small – effect size compared with CRP. Confining analyses to selective serotonin reuptake inhibitor users indicated larger effects of CRP (β_pooled_ = 0.16) and the IMD index (β_pooled_ = 0.20). Baseline IMD features, both separately and combined, did not predict response or remission.

**Conclusions:**

Depressive symptoms of people with more IMD features improved less when treated with antidepressants. However, clinical relevance is limited owing to small effect sizes in inconsistent associations. Whether these patients would benefit more from treatments targeting immunometabolic pathways remains to be investigated.

Antidepressant medication is, besides evidence-based psychotherapies, the first-line treatment choice in depression. Although antidepressants are superior to placebo in terms of efficacy, effect sizes are modest^1^ and about one-third of individuals do not remit, even after multiple attempts.^2^ Considering the complex aetiology of depression and its clinical and biological heterogeneity, depression treatment may be improved by advancing a personalised medicine approach, establishing patient profiles that help to predict treatment success. With the rise of dimensional approaches to the classification of psychiatric disorders, based not only on clinical, but also on biological features,^3^ the immunometabolic depression (IMD) dimension has recently been introduced as a potentially relevant personalised medicine concept. The IMD dimension is hypothesised to reflect a clustering of specific depressive symptoms on the atypical spectrum indicating altered energy homeostasis (e.g. increased appetite/weight, hypersomnia, extreme fatigue and leaden paralysis) and inflammatory and metabolic dysregulations (e.g. increased inflammatory status and disruption of energy-regulating neuroendocrine signalling).^4^ IMD has not been proposed as a new established clinical subtype of depression, but rather as a theoretical dimension to be investigated for its clinical usefulness. Note that the term ‘immune–metabolic’ is used in the immunological literature to refer to the interplay between the immune system and cellular metabolic processes, which is different from what we are referring to with the IMD concept.

## Immunometabolic features and antidepressant treatment outcomes

Elevated inflammation has been demonstrated to contribute to treatment resistance in depression^5^ and – together with metabolic features – to the chronicity of depression in antidepressant users.^6^ Inflammatory biomarkers, such as C-reactive protein (CRP), have previously been suggested to predict poor treatment outcomes with antidepressants,^7^ although findings are mixed.^8^ Similarly, epidemiological evidence shows that obesity (body mass index BMI > 30 kg/m^2^) is more common in patients considered to be treatment-resistant than in those not.^9^ Meta-analyses of clinical studies support the association between higher baseline BMI and both lower response^10^ and remission rates^11^ with first-line antidepressants. Regarding clinical IMD features, although inflammatory markers were not associated with changes in overall depressive symptom severity in the GENDEP trial, small associations were found between inflammation and changes in depressive symptoms related to sleep, weight and appetite.^12^ However, whether severity of a dimensional profile previously labelled ‘atypical energy-related symptoms’ (AES), specifically including hypersomnia, increased appetite and weight, energy loss and leaden paralysis,^13^ predicts responsiveness to antidepressants remains to be elucidated. Moreover, previous studies did not evaluate an IMD index comprising a combination of symptom and biomarker components.

## Aims

This study aims to examine whether three features considered to be indicative of IMD (AES severity, BMI and CRP) at baseline predict antidepressant treatment outcomes by re-analysing and meta-analysing data from four antidepressant treatment trials. Although previous analyses within these trials have been conducted on depressive symptom profiles,^14,15^ BMI ^16–18^ and CRP ^19,20^ as differential predictors of antidepressant treatment outcomes, this study is the first to comprehensively investigate multiple aspects of the IMD concept jointly, regardless of the antidepressant used. It was hypothesised that high levels of AES severity, BMI and CRP would predict poor treatment outcomes. We additionally hypothesised that associations between a composite IMD index based on these features, indicating more severe immunometabolic dysregulations, and antidepressant treatment outcomes would be stronger than for the individual features. Although findings are mixed, a growing body of literature suggests sex differences in antidepressant efficacy. Potential mechanisms underlying these sex differences have been reviewed extensively elsewhere,^21^ but briefly include variations in hormone levels, body fat and liver metabolism affecting the pharmacokinetics of antidepressants, as well as differences in monoamine functioning and medication adherence and side-effects. As previous studies have demonstrated associations between both BMI^18^ and CRP^22^ and antidepressant treatment outcomes only in females but not in males, we explored associations between IMD features and treatment outcomes in males and females separately.

## Method

### Study design and participants

The current study uses data from four studies: International Study to Predict Optimized Treatment in Depression (iSPOT-D); Combining Medications to Enhance Depression Outcomes (CO-MED); Genome-based Therapeutic Drugs for Depression (GENDEP); and Establishing Moderators and Biosignatures of Antidepressant Response in Clinical Care (EMBARC). All four studies included adult participants seeking treatment for depression in primary or psychiatric care settings (see Table 1 for inclusion criteria). Common exclusion criteria were a lifetime history of bipolar disorder, schizophrenia and current substance dependence. Written informed consent was obtained from all participants. All procedures in each study comply with the ethical standards of the relevant national and institutional committees on human experimentation and with the Helsinki Declaration of 1975, as revised in 2008 and were approved by the institutional or ethical review boards of all participating regional centres and clinical sites (iSPOT-D: Copernicus Group IRB, BRA1-08-14, BRA1-09-021; CO-MED: UT Southwestern IRB, 112007-032; GENDEP: South London and Maudsley NHS Trust and Institute of Psychiatry Ethical Committee (Research), 292/03; EMBARC: UT Southwestern IRB, STU 092010-151).

**Table 1:** Sample characteristics for the four individual studies^a^

	Study			
	iSPOT-D	CO-MED	GENDEP	EMBARC
Inclusion criteria				
Age, years	18–65	18–75	≥18	18–86
Diagnosis; instrument	MDD; MINI (DSM-IV)	MDD; clinical interview (DSM-IV)	MDD; SCAN 2.1 (DSM-IV/ICD-10)	MDD; SCID (DSM-IV)
Criteria for current episode	n.a.	≥2 months (recurrent) or ≥2 years (chronic)	Onset at age ≤65	Onset at age ≤30 (early onset), duration >2 years (chronic), or ≥2 recurrences including current (recurrent)
Severity of current episode	At least moderate, HRSD ≥16	At least moderate, HRSD ≥16	At least moderate, DSM-IV/ICD-10	At least mild, QIDS-SR ≥14
Other	n.a.	n.a.	White European ethnicity	n.a.

Total sample, *n*	967	665	793	146
Continuous variables, mean (s.d.)				
Age, years	37.77 (12.61)	42.71 (13.00)	42.45 (11.83)	37.19 (13.77)
Depression severity, norm., baseline	0.55 (0.18)	0.56 (0.16)	0.46 (0.17)	0.46 (0.18)
Depression severity, norm., follow-up	0.36 (0.19)	0.26 (0.19)	0.23 (0.18)	0.40 (0.24)
AES severity	0.27 (0.18)	0.33 (0.19)	0.52 (0.17)	0.48 (0.20)
BMI, kg/m^2^	27.36 (7.13)	31.01 (8.84)	25.53 (4.75)	28.5 (7.94)
Categorical variables, *n* (%)				
Female	548 (57)	452 (68)	500 (63)	102 (70)
Energy loss	602 (62)	512 (77)	734 (93)	131 (90)
Leaden paralysis	n.a.	224 (34)	579 (73)	n.a.
Increased appetite	159 (16)	112 (17)	0 (0)	87 (60)
Increased weight	164 (17)	100 (15)	n.a.	72 (49)
Hypersomnia	163 (17)	75 (11)	0 (0)	45 (31)
Obesity	284 (30)	306 (46)	108 (14)	46 (34)
Response	631 (65)	367 (58)	435 (55)	54 (47)
Remission	357 (37)	255 (38)	309 (39)	45 (39)

CRP sample, *n*	n.a.	166	230	105
Continuous variables, mean (s.d.)				
CRP, mg/L	n.a.	4.39 (7.21)	2.03 (3.24)	2.66 (4.93)
IMD index	n.a.	0.06 (0.73)	0.05 (0.68)	0.07 (0.76)
Categorical variables, *n* (%)				
CRP >1 mg/L	n.a.	110 (66)	98 (43)	60 (57)
CRP >3 mg/L	n.a.	66 (40)	44 (19)	28 (27)

AES, atypical energy-related symptoms; BMI, body mass index; CRP, C-reactive protein; EMBARC, Establishing Moderators and Biosignatures of Antidepressant Response in Clinical Care; GENDEP, Genome-based Therapeutic Drugs for Depression; HRSD, 17-item Hamilton Rating Scale for Depression; IMD, immunometabolic depression; iSPOT-D, International Study to Predict Optimized Treatment in Depression; MDD, major depressive disorder; MINI, Mini International Neuropsychiatric Interview; n.a., not available, norm., normalised using min–max scaling; QIDS-SR, Quick Inventory of Depressive Symptomatology, Self-Report; SCAN, Schedules for Clinical Assessment in Neuropsychiatry interview version; SCID, Structured Clinical Interview for DSM-IV Axis I Disorders.

a. For descriptive purposes, individual AES were recoded into binary variables, with a score of 0 or 1 indicating that the symptom was absent, and a score of 2 or 3 indicating that the symptom was present. As depression severity and its derivative AES severity were measured using different instruments varying in range, normalised means and standard deviations are presented using min–max scaling (i.e. subtracting the minimum and dividing by the range to bring the data between a range of 0 and 1). Data on AES severity were available for 967 patients in iSPOT-D, 665 in CO-MED, 773 in GENDEP and 146 in EMBARC. The number of participants with available data on BMI was 956 in iSPOT-D, 662 in CO-MED, 793 in GENDEP and 134 in EMBARC. An IMD index was constructed based on AES severity, BMI and logarithmically transformed CRP by standardising these variables and taking their mean. The number of participants with available scores on the IMD index was 166 in CO-MED, 226 in GENDEP and 96 in EMBARC.

iSPOT-D (ClinicalTrials.gov identifier: NCT00693849) is an open-label randomised trial undertaken at 17 sites in the USA, The Netherlands, Australia, New Zealand and South Africa.^23^ A total of 1008 participants were randomised to three treatment arms: escitalopram (10–20 mg/day), sertraline (50–200 mg/day) or extended-release venlafaxine (75–225 mg/day) for 8 weeks. Escitalopram and sertraline are selective serotonin reuptake inhibitors (SSRIs) and venlafaxine is a selective serotonin and noradrenaline reuptake inhibitor (SNRI). Response and remission, the primary outcomes of the trial, did not differ between treatment arms.^24^

CO-MED (ClinicalTrials.gov identifier: NCT00590863) is a single-blind randomised placebo-controlled trial conducted in the USA. For a duration of 12 weeks, 665 participants were randomly allocated to three treatment arms: (a) escitalopram (SSRI; 10–20 mg/day) plus placebo, (b) sustained-release bupropion (atypical antidepressant; 150–450 mg/day) plus escitalopram (10–20 mg/day) or (c) extended-release venlafaxine (SNRI; 75–225 mg/day) plus mirtazapine (tetracyclic antidepressant; 15–45 mg/day). Remission, the primary outcome of the trial, did not differ between treatment groups.^25^

GENDEP (EudraCT 2004-001723-38 and ISRCTN03693000) is an open-label partially randomised trial conducted in nine centres in Europe. Of 811 participants, 468 were randomised and 343 were non-randomly allocated. Participants received either 10–30 mg/day escitalopram or 50–150 mg/day nortriptyline for 12 weeks. Nortriptyline is a tricyclic antidepressant with a higher affinity for the noradrenaline transporter than for the serotonin transporter. The primary outcome, depressive symptom severity, did not differ between the two groups.^26^

EMBARC (ClinicalTrials.gov identifier: NCT01407094) is a two-stage placebo-controlled randomised clinical trial conducted across four sites in the USA.^27^ During the first stage of the trial, 296 participants were randomised to sertraline (50–200 mg/day) or placebo for 8 weeks. As the focus of the current study is on active treatments only, data were restricted to 146 patients in the sertraline arm. Participants treated with sertraline did not differ from those receiving placebo on the primary outcome, depression severity, at week 8.^28^

### Outcome measures

The primary outcome of the present study was self-reported depressive symptom severity. The Quick Inventory of Depressive Symptomatology – Self-Report scale (QIDS-SR)^29^ was applied in iSPOT-D and CO-MED to assess depressive symptom severity on all treatment visits after baseline. In GENDEP, in addition to two observer-rated depression rating scales, the self-report Beck Depression Inventory (BDI)^30^ was completed. In EMBARC, however, no self-report scale for depression severity was administered after baseline and we therefore used results from the clinician-rated 17-item Hamilton Rating Scale for Depression (HRSD).^31^ The 16 QIDS-SR items and the 21 BDI items are rated on a four-point scale from 0 to 3. HRSD items are scored either between 0 and 2 or between 0 and 4. Higher total scores indicate higher symptom severity and are therefore less desirable. Maximum total scores varied between the scales and therefore standardised scores were used in the analyses.

Secondary outcomes of the meta-analysis included response and remission. In iSPOT-D, GENDEP and EMBARC, the clinician-rated HRSD was used to indicate response, binary defined as a reduction of at least 50% in depressive symptoms from baseline to the last visit, and remission, defined as an HRSD score ≤7 on the last visit. In CO-MED no clinical instrument was used to establish response and remission, but instead these outcomes were derived from the QIDS-SR. The QIDS-SR does strongly correlate (0.86–0.93) with the HRSD.^2^ Response was defined in CO-MED as a reduction of at least 50% in total QIDS-SR score from baseline to exit and remission was defined as two consecutive QIDS-SR scores with at least one score <6 and one score <8 to rule out false indications of remission based on a single score.

### Assessments of IMD features (AES severity, BMI, CRP)

Severity of atypical energy-related symptoms (AES) was defined as the total score on a symptom profile including: sleeping too much; increased appetite; increased weight; low energy level/fatigue; leaden paralysis.^13^ In CO-MED, these five symptoms were assessed with the Inventory of Depressive Symptomatology, Clinician-Rated (IDS-C).^32^ In iSPOT-D and EMBARC, all but leaden paralysis were assessed with the QIDS-SR. In GENDEP, AES severity was established using the Schedules for Clinical Assessment in Neuropsychiatry interview version 2.1 (SCAN),^33^ in which a clinically trained interviewer rated, among other symptoms: hypersomnia, increased appetite, energy loss and leaden paralysis. The IDS-C, QIDS-SR and SCAN all use a scale from 0 to 3 to indicate the presence and severity of each symptom. A sum score was calculated ranging from 0 to 15 for CO-MED and 0 to 12 for iSPOT-D, GENDEP and EMBARC. Standardised scores were used in the analyses. Data on AES severity were available for 967 participants in iSPOT-D, 665 in CO-MED, 773 in GENDEP and 146 in EMBARC.

BMI was calculated for each participant as weight in kilograms divided by height in metres squared. The number of participants with available data on BMI was 956 in iSPOT-D, 662 in CO-MED, 793 in GENDEP and 134 in EMBARC.

Serum samples of CRP, an acute-phase protein produced in the liver whose circulating concentrations are increased in inflammatory states, were available for the entire sample in EMBARC (*n* = 105), yet only for subsets in GENDEP (*n* = 230) and CO-MED (*n* = 166) and not in iSPOT-D. CRP was derived from antecubital venous blood sampling using a high-sensitivity immunoturbidimetry assay (Cormay, Lublin, Poland) in GENDEP,^19^ a multiplexed immunoassay (Bio-Rad Laboratory, Hercules, Californai, USA) in CO-MED^20^ and an enzyme-linked immunosorbent assay (ELISA, EMD Millipore, Catalog #CYT298) in EMBARC.^22^ As the distributions of CRP were skewed, a natural logarithm transformation was applied, resulting in approximately normal distributions. Participants with CRP values >10 were not excluded because various factors, such as age, sex, socioeconomic status, ethnicity, medication use, BMI and lifestyle factors can affect circulating CRP levels, which may not always reflect underlying pathology.^34^

AES severity, BMI and CRP, as well as demographic information (age and sex at birth), were all recorded before the start of treatment. An IMD index was constructed based on AES severity, BMI and logarithmically transformed CRP by standardising these variables and taking their mean. Higher scores on this index are indicative of a greater IMD burden. No IMD index was computed for iSPOT-D, owing to a lack of CRP data. The number of participants with available scores on the IMD index was 166 in CO-MED, 226 in GENDEP and 96 in EMBARC.

### Statistical analyses

Statistical analyses were carried out using R Version 4.2.1 for Windows. We used standardised scores (*Z*-scores) in the regression analyses in each study separately for the predictors AES severity, BMI and CRP, as well as for the primary outcome depression severity, to obtain normalised coefficients independent of measurement units. Pearson correlations between baseline IMD features were first computed within each study and then meta-analysed.

Consistent with previous GENDEP and CO-MED analyses,^19,20^ linear mixed models with a random intercept for the individual were used to examine whether baseline IMD features predicted change in depression symptom severity within each study separately. For secondary binary outcomes, logistic regression analyses were performed. Covariates included age, sex, baseline depression severity and time (linear and quadratic) for linear mixed models and age and sex for logistic regression models. No issues regarding collinearity between AES severity (predictor) and baseline depression severity (covariate) were found, as indicated by correlation coefficients ranging from 0.005 to 0.41 and a maximum variance inflation factor (VIF) value of 1.23 (i.e. below the threshold of 2.5). Main effects within the individual studies were pooled using random-effects meta-analyses. The Q-test was performed to assess the heterogeneity in the variances of the effect sizes, complemented by the *I*^2^ statistic.^35^

To explore whether effects on the primary outcome depressive symptom severity depend on which antidepressant was used predictor × treatment interactions were added to the models and tested within CO-MED, iSPOT-D and GENDEP separately. No interactions were tested in EMBARC as this trial included only one active treatment arm. In total, 22 treatment comparisons were tested and *P*-values were together adjusted for multiple testing using the false discovery rate (FDR) correction.

We performed similar analyses including individual continuous atypical, energy-related symptom scores as predictors of treatment outcomes. Additionally, we conducted some *post hoc* explorative analyses. First, we explored whether categorically defined obesity (BMI >30 kg/m^2^) predicted treatment outcomes. Second, we assessed two categorical definitions of inflammation based on CRP >1 mg/L (indicative of elevated CRP), as well as CRP >3 mg/L (indicative of low-grade inflammation).^36^ Third, meta-analyses on the primary outcome were confined to SSRI users only, as this was the only antidepressant class that was comparable across treatment arms. Fourth, meta-analyses were performed in subsets of only males and only females. All analyses were conducted on an intention-to-treat basis. Two-tailed statistical tests were used and statistical significance was set at *P <* 0.05, unless otherwise specified.

## Results

Characteristics of each study are summarized in Table 1. Pooled analyses of bivariate correlations between baseline IMD features showed that AES severity was significantly and positively correlated with BMI (*n* = 2493, *r*_pooled_ = 0.17, *P* = 5.01 × 10^−6^, 95% CI 0.10–0.25), but not with CRP (*n* = 497, *r*_pooled_ = 0.08, *P* = 0.166, 95% CI −0.04 to 0.02). In addition, pooled analyses revealed the expected positive correlation between BMI and CRP (*N* = 492, *r*_pooled_ = 0.45, *P* = 6.87 × 10^−8^, 95% CI 0.33–0.62).

### Baseline IMD features as predictors of depressive symptom severity

**Fig. 1:**
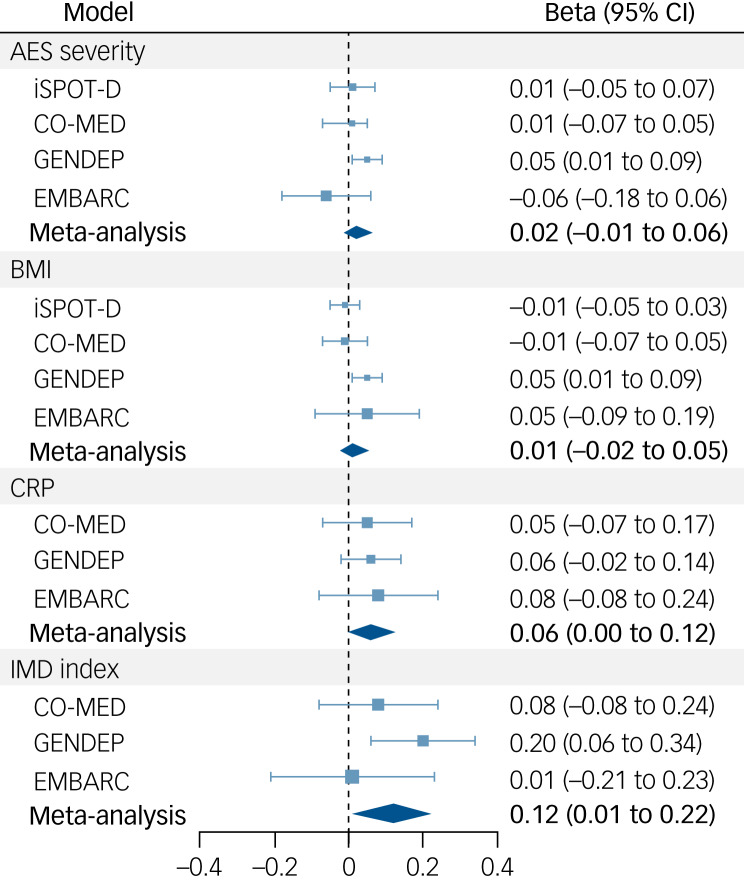
Baseline immunometabolic depression (IMD) features (individually and combined into an IMD index) predicting depressive symptom severity over the course of treatment with antidepressants for the iSPOT-D, CO-MED, GENDEP and EMBARC studies. AES, atypical energy-related symptoms; BMI, body mass index; CO-MED, Combining Medications to Enhance Depression Outcomes; CRP, C-reactive protein levels; EMBARC, Establishing Moderators and Biosignatures of Antidepressant Response in Clinical Care; GENDEP, Genome-based Therapeutic Drugs for Depression; iSPOT-D, International Study to Predict Optimized Treatment in Depression.

A higher IMD index significantly predicted a smaller reduction in depressive symptom severity over the course of treatment (*n* = 372, β_pooled_ = 0.12, s.e. = 0.12, *P =* 0.031, 95% CI 0.01–0.22) with low heterogeneity (*I*^2^ *=* 23.91%) (Table 2 and Fig. 1). Regarding individual IMD features, AES severity and BMI did not predict depressive symptom severity. Although no significant association was found between CRP and depressive symptom severity within individual studies, a significant but small positive association was found in the meta-analysis (*n* = 376, β_pooled_ = 0.06, s.e. = 0.03, *P =* 0.049, 95% CI 0.0001–0.12). This indicates that participants with higher baseline CRP showed less improvement in depression symptom severity. Regarding differential treatment response, none of the IMD feature × treatment interaction effects were significant, except for a significant CRP × treatment interaction effect (*P*_FDR_ *=* 0.004) in GENDEP, which has previously been reported^19^ (Supplementary Table 1, available at https://doi.org/10.1192/bjp.2023.148).

**Table 2:** Baseline features of immunometabolic depression (IMD) predicting depressive symptom severity over the course of treatment^a^

Predictor	Study	*n*	β^b^ (s.e.)	*P*	95% CI
AES severity	iSPOT-D	967	0.01 (0.03)	0.572	−0.05 to 0.07
	CO-MED	633	0.01 (0.03)	0.615	−0.07 to 0.05
	GENDEP	745	0.05 (0.02)	0.053	0.01 to 0.09
	EMBARC	138	−0.06 (0.06)	0.320	−0.18 to 0.06
	Meta-analysis	2483	0.02 (0.02)	0.222	−0.01 to 0.06
	Heterogeneity	*Q*(3) = 4.06, *P* = 0.255, *I*^2^ = 23.28%			

BMI	iSPOT-D	956	−0.01 (0.02)	0.598	−0.05 to 0.03
	CO-MED	630	−0.01 (0.03)	0.595	−0.07 to 0.05
	GENDEP	762	0.05 (0.02)	0.039*	0.01 to 0.09
	EMBARC	128	0.05 (0.07)	0.448	−0.09 to 0 to 19
	Meta-analysis	2476	0.01 (0.02)	0.421	−0.02 to 0.05
	Heterogeneity	*Q*(3) = 5.57, *P* = 0.135, *I*^2^ = 48.81%			

CRP	CO-MED	157	0.05 (0.06)	0.337	−0.07 to 0.17
	GENDEP	219	0.06 (0.04)	0.157	−0.02 to 0.14
	EMBARC	100	0.08 (0.08)	0.331	−0.08 to 0.24
	Meta-analysis	376	0.06 (0.03)	0.049*	0.0001 to 0.12
	Heterogeneity	*Q*(2) = 0.09, *P* = 0.956, *I*^2^ = 0.00%			

IMD index	CO-MED	157	0.08 (0.08)	0.280	−0.08 to 0.24
	GENDEP	215	0.20 (0.07)	0.003**	0.06 to 0.34
	EMBARC	92	0.01 (0.11)	0.910	−0.21 to 0.23
	Meta-analysis	372	0.12 (0.05)	0.031*	0.01 to 0.22
	Heterogeneity	*Q*(2) = 2.55, *P* = 0.279, *I*^2^ = 23.91%			

AES, atypical energy-related symptoms; BMI, body mass index; CO-MED, Combining Medications to Enhance Depression Outcomes; CRP, C-reactive protein; EMBARC, Establishing Moderators and Biosignatures of Antidepressant Response in Clinical Care; GENDEP, Genome-based Therapeutic Drugs for Depression; iSPOT-D, International Study to Predict Optimized Treatment in Depression.

a. Linear mixed models with a random intercept for the individual were adjusted for age, sex, time (linear and quadratic) and baseline depression severity. Random effects meta-analyses were used to pool the results. Depression severity was measured over multiple time-points between baseline and exit (iSPOT-D: week 2, 4, 6 and 8; CO-MED: week 1, 2, 4, 6, 8 and 12, GENDEP: week 1–12, EMBARC: week 1–8).

b. β is the normalised regression coefficient for the main effect of IMD feature or the IMD index (based on AES severity, BMI and log of CRP) on depression severity.

*P < 0.05, **P < 0.01.

### Baseline IMD features as predictors of response and remission

**Fig. 2:**
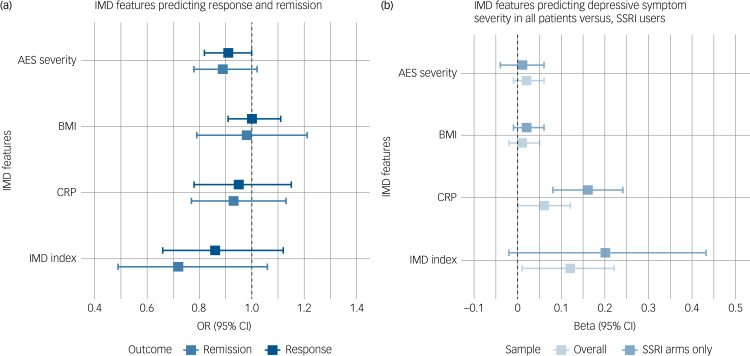
(a) Meta-analyses on secondary outcomes. (b) Sensitivity analyses on depressive symptom severity in all patients versus selective serotonin reuptake inhibitor (SSRI) users only. IMD, immunometabolic depression; AES, atypical energy-related symptoms; BMI, body mass index; CRP, C-reactive protein levels.

Among the total sample, 58% (1515/2630) responded and 37% (979/2630) achieved remission. Meta-analyses did not indicate significant effects of baseline IMD features on either treatment response or remission (Fig. 2(a) and Supplementary Table 2). Substantial heterogeneity among study findings was found regarding the effect of BMI on remission, as indicated by the Q-test (*P* = 0.001) and an *I*^2^ of 78.59%. Participants with higher BMI were more likely to remit in iSPOT-D, but less likely to remit in GENDEP. No association between baseline BMI and remission was found in the other two studies, nor in the meta-analysis pooling the results.

### Individual AES as predictors of treatment outcomes

Of the individual AES, meta-analyses showed that leaden paralysis was a significant predictor of worse treatment outcomes: higher leaden paralysis at baseline was associated with less reduction in depressive symptom severity (β_pooled_ = 0.05, s.e. = 0.02, *P* = 0.001, 95% CI 0.02–0.09) and a lower likelihood to respond (OR_pooled_ = 0.85, *P* = 0.001, 95% CI 0.76–0.94) or achieve remission (OR_pooled_ = 0.80, *P* = 0.016, 95% CI 0.66–0.96). Higher energy loss at baseline was associated with less reduction in depressive symptom severity (β_pooled_ = 0.04, s.e. = 0.02, *P* = 0.044, 95% CI 0.001–0.08). The other AES were not associated with any of the outcomes (Supplementary Table 3, Supplementary Fig 1).

### *Post hoc* analyses

Among the total sample, 28% (744/2630) were classified as obese (BMI >30 kg/m^2^) and, among the sample with available CRP data, 53% (268/501) and 28% (138/501) had CRP levels above 1 and 3 respectively. Obesity did not predict any of the treatment outcomes (Supplementary Table 4). However, there was large between-study heterogeneity (*I*^2^ ranging from 64.62 to 75.72%). Participants with CRP > 1 improved significantly less in depressive symptom severity compared with those with CRP ≤ 1 (β_pooled_ = 0.13, s.e. = 0.06, *P =* 0.041, 95% CI 0.01–0.26). Using a cut-off of 3 resulted in a similar pooled regression coefficient, although this effect was non-significant (β_pooled_ = 0.13, s.e. = 0.07, *P =* 0.090, 95% CI −0.02 to 0.28). Categorical definitions of inflammation did not predict either treatment response or remission (Supplementary Table 4).

The pooled regression coefficient for the IMD index was higher when including SSRI arms only, compared with that in the main analysis (*n* = 242, β_pooled_ = 0.20, s.e. = 0.12, *P =* 0.079, 95% CI −0.02 to 0.43). Although the regression coefficients in all point in the same direction, heterogeneity was substantial (*I*^2^ = 70.24%) and significant (*P* = 0.029). The pooled effect of baseline CRP was also higher and remained significant (*n* = 253, β_pooled_ = 0.16, s.e. = 0.04, *P =* 0.0002, 95% CI 0.08–0.24, *I^2^* *=* 1.93%). Similar results as in the main analyses were returned regarding the effects of AES severity and BMI (Fig. 2(b) and Supplementary Table 5).

For males, the mean age ranged from 37.55 years (s.d. = 12.59) in EMBARC to 45.12 years (s.d. = 12.34) in CO-MED, and the mean BMI ranged from 26.26 (s.d. = 3.68) in GENDEP to 29.87 (s.d. = 7.47) in CO-MED. For females, the mean age ranged from 37.04 years (s.d. = 14.30) in EMBARC to 41.62 years (s.d. = 10.81) in GENDEP, and the mean BMI ranged from 25.30 (s.d. = 5.65) in GENDEP to 31.54 (s.d. = 9.37) in CO-MED. Pooled analyses on subsets of males and females only (Supplementary Table 6) revealed that higher IMD index was associated with less improvement in depressive symptom severity in females (*n* = 313, β_pooled_ = 0.14, s.e. = 0.06, *P =* 0.013, 95% CI 0.03–0.26), but not in males (*n* = 151, β_pooled_ = 0.07, s.e. = 0.12, *P =* 0.545, 95% CI −0.16 to 0.30). Although the effect of AES severity on depressive symptom severity change was not significant in the pooled analysis including the entire sample, higher AES severity was associated with less reduction in depressive symptoms in males (*n* = 933, β_pooled_ = 0.06, s.e. = 0.02, *P* = 0.019, 95% CI 0.01–0.10).

## Discussion

This study investigated three IMD features (AES severity, BMI and CRP), both singularly and cumulatively, as putative predictors of antidepressant treatment outcomes, using data from four antidepressant treatment trials. Depressive symptoms of patients with more IMD features at baseline improved slightly less over the course of antidepressant treatment, mostly driven by CRP. Associations with response and remission were not confirmed. As effect sizes were small and IMD features were not consistently related to outcomes, the clinical relevance of these findings is limited.

### Predictors of treatment outcome in the total sample

The heterogeneity of depression in symptom profiles and underlying biological mechanisms holds promise for future developments in personalised psychiatry. One of these promises is that clinically and/or biologically more homogeneous depression dimensions, such as the proposed IMD dimension, could predict treatment outcomes to an extent that they could inform treatment decisions in routine clinical practice. Our results did show that higher CRP and higher scores on a cumulative IMD index, also including BMI and AES severity, at baseline predicted smaller reductions in depressive symptom severity during treatment with antidepressants. The IMD index showed larger effect size than CRP (0.12 *v.* 0.06), indicating cumulative effects not visible at individual (BMI or AES severity) level. Sex-specific analyses indicated that the association between IMD index and depression severity change was present only in females. Factors such as hormonal contraceptives and menstrual cycle potentially influence antidepressant effects through alterations in drug metabolism and monoamine regulation.^21^ These findings highlight the importance of specifically addressing sex differences when studying depression treatment. However, although statistically significant, the clinical significance of these findings is unlikely, given the small effect sizes and lack of evidence for associations with end-points of clinical interest, such as remission.

### Predictors of treatment outcome in SSRI users only

Confining analyses to SSRI users only, effects of the CRP and the IMD index became larger, yet still of small magnitude (effect sizes 0.16 and 0.20 respectively). This finding aligns with previous evidence indicating that depressed patients with low-grade inflammation benefit less from predominantly serotonergic antidepressants compared with noradrenergic or dopaminergic antidepressants (as reviewed in^37^). SSRIs work by blocking the reuptake of serotonin, leading to increased serotonin in the synapse. However, inflammatory cytokines have been found to disrupt this process by increasing the expression of monoamine transporters through the activation of mitogen-activated protein kinase pathways.^38^ This results in higher serotonin reuptake at the presynaptic terminal and reduced availability of serotonin in the synapse. Moreover, increased inflammation is found to promote the breakdown of tryptophan through the kynurenine pathway. This not only leads to reduced availability of tryptophan for serotonin synthesis, but also contributes to glutamate imbalances. Glutamate imbalances have been associated with reduced neuroplasticity, which is essential for optimal efficacy of SSRIs.^38^

The current findings are contrary to those of previous meta-analyses indicating a link between higher inflammation for some – but not all – inflammatory markers and BMI at baseline and lower response or remission with antidepressant treatment.^7,10,11^ However, it is important to note that in these meta-analyses, significant heterogeneity among study findings was reported that could be explained by type of treatment (SSRI monotherapy versus combined therapy), sample (out-patient versus in-patient) and type of assay or serum versus plasma sample. Our findings do match those observed in other antidepressant treatment studies stratifying patients along other potentially treatment-relevant dimensions. Besides IMD, dimensions based on childhood trauma,^39^ hypothalamic–pituitary–adrenal axis activity,^40^ melancholic^41^ or anxious features,^42^ have also been unable to clearly differentiate patients benefitting from antidepressants and those not. More success has been achieved in the case of electroconvulsive therapy (ECT), in which psychotic and catatonic features are well-established predictors of remission.^43^ However, effect sizes are generally much lower for antidepressants than for ECT (i.e. 2 *v.* 9.7 points difference in total HRSD scores between treatment and placebo^1,44^). Considering that a great portion of the variance in treatment outcomes is generally explained by non-specific therapeutic factors (e.g. alliance, adherence, expectancy effects)^45^ and error variance, the scope for clinical or biological phenotypes may be only limited.

### Further research

Although the value of depression dimensions such as IMD in deciding whether or not to consider regular antidepressant treatment is limited, they may still be useful to distinguish patients in need of specific (add-on) treatments directly addressing underlying mechanisms involved in these pathologies, for example anti-inflammatory medication in IMD.^46^ To optimise depression treatment, it has been proposed that clinical trials are needed to develop more targeted treatments. These trials should (a) screen participants for the pathology of interest using symptoms or biomarkers associated with the pathophysiologic mechanism being targeted, (b) verify whether the treatment indeed affected the target and (c) evaluate treatment effects using outcome variables reflecting the biology/symptoms involved.^47^

Several questions related to personalised treatment for IMD in its current conceptualisation remain unanswered at present. For example, further work is required to establish whether features of IMD put individuals at greater risk for side-effect burden. Additionally, although our study indicated that IMD features prior to treatment with antidepressants do not predict clinical improvement in depression, their effect on physical health outcomes, including metabolic and immune indicators, is poorly understood. Moreover, our analyses are heterogeneous in terms of antidepressant classes. It is possible that individuals with IMD characteristics benefit more from some antidepressant (class) than another. In GENDEP, participants with high levels of inflammation benefitted from the noradrenergic nortriptyline more than from SSRI monotherapy.^19^ Findings from the iSPOT-D trial also suggested better outcomes for venlafaxine-XR compared with SSRI monotherapy in participants with the highest BMI.^18^ However, in GENDEP, higher BMI predicted poor response to nortriptyline but did not significantly influence response to escitalopram.^17^ This could explain high heterogeneity among study findings in the analyses on BMI. Previous findings from the CO-MED trial suggested better outcomes for a bupropion–SSRI combination compared with SSRI monotherapy in participants with extreme obesity (BMI ≥ 35 kg/m^2^)^16^ and higher baseline CRP.^20^ More studies are needed to further replicate these findings. Finally, it remains to be investigated whether patients with IMD features benefit more from treatments targeting immunometabolic pathways, for example anti-inflammatory treatment or lifestyle interventions based on diet, energy restriction or increased physical activity.

### Limitations

Our study has several limitations. First, our findings are limited to selected proposed indicators of IMD. CRP is a non-specific marker of inflammation and associations with antidepressant treatment response have been inconclusive.^8^ Moreover, although well-known as a marker of inflammation, CRP may also have some anti-inflammatory effects.^48^ Future treatment studies should therefore incorporate more diverse indicators of inflammation, as done recently in GENDEP.^12^ Nevertheless, CRP is an easily obtained measure and suitable for use in the clinic. Second, the validity of BMI as a measure of metabolic health has been questioned.^49^ Third, owing to limited data availability on CRP, the sample sizes in the pooled analyses on CRP and the IMD index were relatively small, reducing statistical power. Fourth, not all studies used the same scale to measure depressive symptom severity, which impeded harmonisation of data on the primary outcome. Fifth, since the IMD index is a first attempt to combine multiple IMD features into a single score, its reliability and validity require further investigation. Lastly, owing to limited data availability or differences in assessment across trials we cannot rule out confounding by inflammatory diseases, anti-inflammatory medications, hormonal contraceptive use and menstrual phase.

## Supporting information

Vreijling et al. supplementary material 1 Vreijling et al. supplementary material

Vreijling et al. supplementary material 2 Vreijling et al. supplementary material

Vreijling et al. supplementary material 3 Vreijling et al. supplementary material

## Data Availability

CO-MED and EMBARC data can be found using a National Institute of Mental Health (NIMH) data request and the following study identification numbers: NCT00590863; NCT01407094. The iSPOT-D data-set analysed in the current study will be made available from the academic principal investigator L.M.W. on reasonable request after approval of a proposal. Reasonable requests will also require the permission of the study sponsor. Data from the GENDEP study are available by application to cathryn.lewis@kcl.ac.uk at King's College London.
