# Supplementary material for: Features of immunometabolic depression as predictors of antidepressant treatment outcomes: pooled analysis of four clinical trials
Source: Br J Psychiatry. 2024 Mar;224(3):89–97. doi: 10.1192/bjp.2023.148 (PMC10884825; doi:10.1192/bjp.2023.148)
Supplement: Vreijling et al. supplementary material 1 — Vreijling et al. supplementary material [file S0007125023001484sup001.pdf]

Meta-analyses on the effects of individual atypical, energy-related symptoms and atypical, energy-related symptom severity (AES severity) at baseline on antidepressant treatment outcomes: results from iSPOT-D, CO-MED, GENDEP and EMBARC

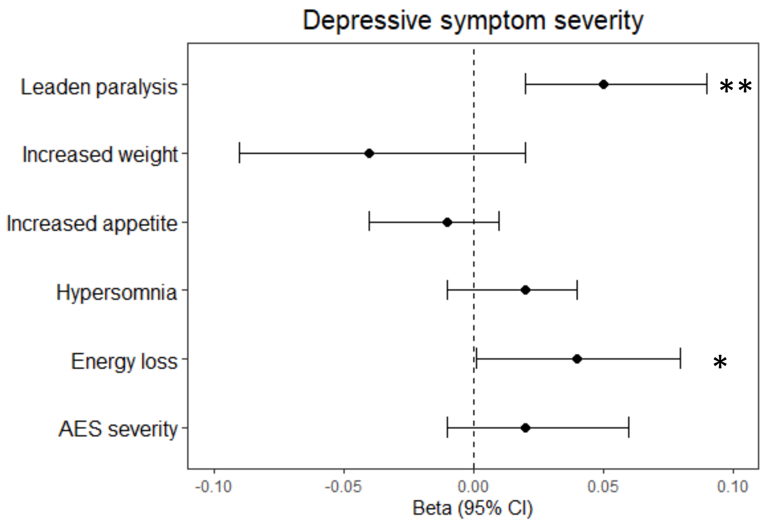

*Figure 1. Individual atypical, energy-related symptoms and AES severity as predictors of depressive symptom severity during treatment. **p <.01 *p <.05.*

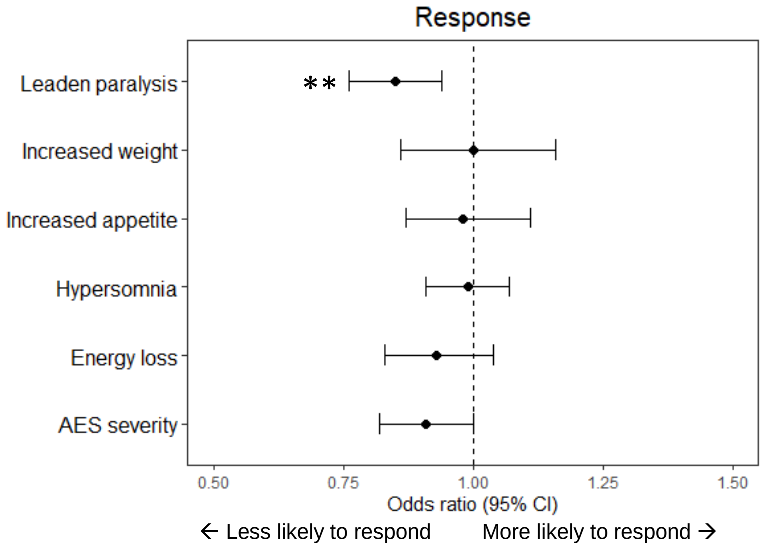

Figure 2. Individual atypical, energy-related symptoms and AES severity as predictors of treatment response.

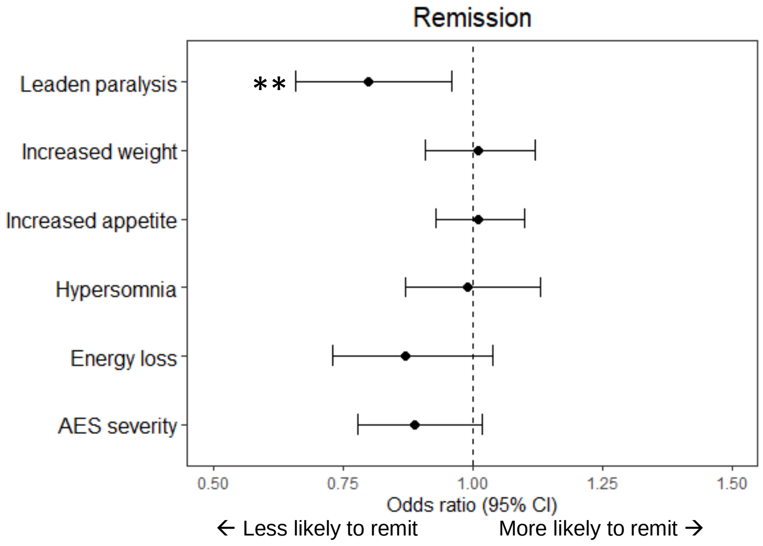

Figure 3. Individual atypical, energy-related symptoms and AES severity as predictors of remission.
