# Supplementary material for: Features of immunometabolic depression as predictors of antidepressant treatment outcomes: pooled analysis of four clinical trials
Source: Br J Psychiatry. 2024 Mar;224(3):89–97. doi: 10.1192/bjp.2023.148 (PMC10884825; doi:10.1192/bjp.2023.148)
Supplement: Vreijling et al. supplementary material 2 — Vreijling et al. supplementary material [file S0007125023001484sup002.docx]

**SUPPLEMENTARY MATERIAL**

Supplementary Table 1: IMD feature-by-treatment interaction effects in the analyses on depressive symptom severity.

| **iSPOT-D** |  |  |  |  |  |
| --- | --- | --- | --- | --- | --- |
| **IMD feature** | **Comparison** | **β** | **SE** | ***p*** | ***p*_FDR_** |
| AES | Ecitalopram vs. Venlafaxine | -0,05 | 0,06 | 0,393 | 0.625 |
| AES | Sertraline vs. Venlafaxine | -0,12 | 0,05 | **0,024*** | 0.179 |
| AES | Sertraline vs. Escitalopram | 0,07 | 0,05 | 0,174 | 0.635 |
| BMI | Ecitalopram vs. Venlafaxine | 0,05 | 0,06 | 0,337 | 0.635 |
| BMI | Sertraline vs. Venlafaxine | -0,01 | 0,06 | 0,883 | 0.884 |
| BMI | Sertraline vs. Escitalopram | 0,06 | 0,05 | 0,248 | 0.635 |
| **CO-MED** |  |  |  |  |  |
| **IMD feature** | **Comparison** | **β** | **SE** | ***p*** | ***p*_FDR_** |
| AES | Ecitalopram vs. Buproprion + escitalopram | 0,01 | 0,06 | 0,875 | 0.884 |
| AES | Venlafaxine + mirtazepine vs. Buproprion + escitalopram | 0,04 | 0,06 | 0,519 | 0.714 |
| AES | Venlafaxine + mirtazepine vs. Escitalopram | 0,03 | 0,07 | 0,646 | 0.789 |
| BMI | Ecitalopram vs. Buproprion + escitalopram | 0,05 | 0,06 | 0,424 | 0.635 |
| BMI | Venlafaxine + mirtazepine vs. Buproprion + escitalopram | -0,06 | 0,07 | 0,373 | 0.635 |
| BMI | Venlafaxine + mirtazepine vs. Escitalopram | -0,11 | 0,06 | 0,067 | 0.369 |
| CRP | Ecitalopram vs. Buproprion + escitalopram | 0,16 | 0,14 | 0,247 | 0.635 |
| CRP | Venlafaxine + mirtazepine vs. Buproprion + escitalopram | 0,14 | 0,13 | 0,296 | 0.635 |
| CRP | Venlafaxine + mirtazepine vs. Escitalopram | -0,02 | 0,13 | 0,884 | 0.884 |
| IMD | Ecitalopram vs. Buproprion + escitalopram | 0,15 | 0,19 | 0,433 | 0.635 |
| IMD | Venlafaxine + mirtazepine vs. Buproprion + escitalopram | 0,05 | 0,20 | 0,791 | 0.884 |
| IMD | Venlafaxine + mirtazepine vs. Escitalopram | -0,10 | 0,18 | 0,580 | 0.750 |
| **GENDEP** |  |  |  |  |  |
| **IMD feature** | **Comparison** | **β** | **SE** | **p** | ***p*_FDR_** |
| AES | Escitalopram vs. Nortriptyline | -0,06 | 0,05 | 0,198 | 0.635 |
| BMI | Escitalopram vs. Nortriptyline | 0,04 | 0,05 | 0,391 | 0.635 |
| CRP | Escitalopram vs. Nortriptyline | -0,32 | 0,08 | **0,0002***** | 0.004** |
| IMD | Escitalopram vs. Nortriptyline | -0,35 | 0,13 | **0,006**** | 0.067 |
| Abbreviations: AES = atypical, energy-related symptoms, BMI = body mass index, CRP = C-reactive protein logarithmically transformed; *p*_FDR_ = *p-value* adjusted for multiple testing using false discovery rate correction. IMD = immuno-metabolic depression. SE = Standard error.  Note. Linear mixed models with a random intercept for the individual, adjusted for age, sex, time (linear and quadratic), baseline depression severity, IMD feature and treatment. An IMD index was constructed based on AES severity, BMI and logarithmically transformed CRP by standardizing these variables and taking their mean. | | | | | |
| ***p <.001 **p <.01 *p <.05 | |  |  |  |  |

Supplementary Table 2. Baseline IMD features predicting response and remission

|  |  |  | **Response** | | | |  | **Remission** | | | |
| --- | --- | --- | --- | --- | --- | --- | --- | --- | --- | --- | --- |
| **Predictor** |  | **Study** | **N** | **OR** | ***p*** | **CI** |  | **N** | **OR** | ***p*** | **CI** |
| AES severity |  | iSPOT-D | 967 | 0.98 | 0.749 | 0.85,1.12 |  | 967 | 1.02 | 0.733 | 0.89,1.17 |
|  |  | CO-MED | 633 | 0.95 | 0.564 | 0.81,1.11 |  | 665 | 0.84 | 0.040* | 0.72,0.99 |
|  |  | GENDEP | 773 | 0.81 | 0.006** | 0.71,0.93 |  | 773 | 0.79 | 0.002** | 0.67,0.92 |
|  |  | EMBARC | 114 | 0.88 | 0.496 | 0.62,1,26 |  | 114 | 0.95 | 0.789 | 0.66,1.38 |
|  |  | **Meta-analysis** | **2487** | **0.91** | **0.050** | **0.82,1.00** |  | **2519** | **0.89** | **0.089** | **0.78,1.02** |
|  |  | Heterogeneity | *Q*(3) = 4.20, *p* = 0.241, *I^2^ =* 37.39% | | | |  | *Q*(3) = 6.43, *p* = 0.092, *I^2^ =* 54.36% | | | |
| BMI |  | iSPOT-D | 956 | 1.08 | 0.282 | 0.94,1.24 |  | 956 | 1.24 | 0.001** | 1.09,1.43 |
|  |  | CO-MED | 630 | 1.00 | 0.953 | 0.85,1.17 |  | 662 | 0.96 | 0.574 | 0.81,1.11 |
|  |  | GENDEP | 793 | 0.97 | 0.715 | 0.85,1.11 |  | 793 | 0.82 | 0.013* | 0.70,0.96 |
|  |  | EMBARC | 105 | 0.72 | 0.118 | 0.47,1.07 |  | 105 | 0.91 | 0.640 | 0.60,1.37 |
|  |  | **Meta-analysis** | **2484** | **1.00** | **0.983** | **0.91, 1.11** |  | **2516** | **0.98** | **0.865** | **0.79,1.21** |
|  |  | Heterogeneity | *Q*(3) = 3.84, *p* = 0.280, *I^2^ =* 0.02% | | | |  | *Q*(3) = 16.02, *p* = 0.001, *I^2^ =* 78.59% | | | |
| CRP |  | CO-MED | 157 | 0.80 | 0.181 | 0.57,1.11 |  | 166 | 0.96 | 0.818 | 0.70,1.31 |
|  |  | GENDEP | 230 | 1.06 | 0.694 | 0.80,1.38 |  | 230 | 0.86 | 0.304 | 0.65,1.13 |
|  |  | EMBARC | 83 | 0.97 | 0.902 | 0.61,1.55 |  | 83 | 1.10 | 0.700 | 0.68,1.75 |
|  |  | **Meta-analysis** | **470** | **0.95** | **0.603** | **0.78,1.15** |  | **479** | **0.93** | **0.461** | **0.77,1.13** |
|  |  | Heterogeneity | *Q*(2) = 1.64, *p* = 0.440, *I^2^ =* 3.61% | | | |  | *Q*(2) = 0.84, *p* = 0.657, *I^2^ =* 0% | | | |
| IMD index |  | CO-MED | 157 | 0.80 | 0.341 | 0.51,1.26 |  | 166 | 0.75 | 0.217 | 0.48,1.19 |
|  |  | GENDEP | 226 | 0.91 | 0.625 | 0.61,1.34 |  | 226 | 0.53 | 0.006** | 0.34,0.83 |
|  |  | EMBARC | 76 | 0.86 | 0.624 | 0.46,1.60 |  | 76 | 1.09 | 0.797 | 0.58,2.03 |
|  |  | **Meta-analysis** | **459** | **0.86** | **0.272** | **0.66,1.12** |  | **468** | **0.72** | **0.096** | **0.49,1.06** |
|  |  | Heterogeneity | *Q*(2) = 0.179, *p* = 0.915, *I^2^ =* 0% | | | |  | *Q*(2) = 3.47, *p* = 0.091, *I^2^ =* 41.09% | | | |

*Abbreviations:* OR = odds ratio. *Notes:* Logistic regression models were adjusted for age and sex. Random effects meta-analyses were used to pool the results. Response was defined as a reduction of at least 50% in HRSD (iSPOT-D, GENDEP, EMBARC) or QIDS (CO-MED) score from baseline to exit. Remission was defined as HRSD ≤ 7 (iSPOT, GENDEP, EMBARC) or two consecutive QIDS scores of at least one score < 6 and one score < 8 (CO-MED).

Supplementary Table 3: Individual atypical, energy-related symptoms (AES) as predictors of treatment outcomes

| \|  \|  \| **Depressive symptom severity** \| \| \| \| \|  \|  \| **Response** \| \| \| \| **Remission** \| \| \|  \| \| --- \| --- \| --- \| --- \| --- \| --- \| --- \| --- \| --- \| --- \| --- \| --- \| --- \| --- \| --- \| --- \| --- \| \| **Predictor** \| **Study** \| **β** \| **SE** \| **p** \| **95% CI** \| \|  \| **OR** \| **p** \| **95% CI** \| \|  \| **OR** \| **p** \| **95% CI** \| \| \| Hypersomnia \| COMED \| 0.004 \| 0.03 \| 0.893 \| -0.05 \| 0.06 \|  \| 0.92 \| 0.311 \| 0.78 \| 1.08 \|  \| 0.83 \| 0.031* \| 0.70 \| 0.98 \| \|  \| iSPOT \| 0.03 \| 0.02 \| 0.111 \| -0.05 \| 0.04 \|  \| 1.08 \| 0.250 \| 0.95 \| 1.24 \|  \| 1.10 \| 0.149 \| 0.97 \| 1.23 \| \|  \| GENDEP \| 0.004 \| 0.02 \| 0.848 \| -0.04 \| 0.05 \|  \| 0.97 \| 0.710 \| 0.84 \| 1.12 \|  \| 1.05 \| 0.480 \| 0.91 \| 1.21 \| \|  \| EMBARC \| 0.05 \| 0.06 \| 0.428 \| -0.07 \| 0.17 \|  \| 0.94 \| 0.730 \| 0.65 \| 1.35 \|  \| 0.95 \| 0.800 \| 0.66 \| 1.38 \| \|  \| **Meta-analysis** \| **0.02** \| **0.01** \| **0.139** \| **-0.01** \| **0.04** \|  \| **0.99** \| **0.829** \| **0.91** \| **1.07** \|  \| **0.99** \| **0.906** \| **0.87** \| **1.13** \| \| Increased appetite \| COMED \| -0.05 \| 0.03 \| 0.081 \| -0.10 \| 0.01 \|  \| 1.14 \| 0.130 \| 0.96 \| 1.34 \|  \| 1.06 \| 0.442 \| 0.61 \| 0.85 \| \|  \| GENDEP \| -0.0002 \| 0.02 \| 0.995 \| -0.05 \| 0.05 \|  \| 0.96 \| 0.621 \| 0.84 \| 1.11 \|  \| 1.04 \| 0.587 \| 0.90 \| 1.20 \| \|  \| iSPOT \| -0.01 \| 0.02 \| 0.807 \| -0.05 \| 0.04 \|  \| 0.95 \| 0.469 \| 0.83 \| 1.09 \|  \| 0.97 \| 0.613 \| 0.85 \| 1.10 \| \|  \| EMBARC \| -0.03 \| 0.06 \| 0.571 \| -0.15 \| 0.08 \|  \| 0.72 \| 0.099 \| 0.49 \| 1.06 \|  \| 0.82 \| 0.319 \| 0.56 \| 1.21 \| \|  \| **Meta-analysis** \| **-0.01** \| **0.01** \| **0.341** \| **-0.04** \| **0.01** \|  \| **0.98** \| **0.756** \| **0.87** \| **1.11** \|  \| **1.01** \| **0.807** \| **0.93** \| **1.10** \| \| Increased weight \| COMED \| -0.05 \| 0.03 \| 0.062 \| -0.10 \| 0.002 \|  \| 1.10 \| 0.240 \| 0.94 \| 1.30 \|  \| 1.04 \| 0.608 \| 0.89 \| 1.22 \| \|  \| iSPOT \| -0.002 \| 0.02 \| 0.943 \| -0.05 \| 0.04 \|  \| 0.90 \| 0.128 \| 0.79 \| 1.03 \|  \| 0.98 \| 0.787 \| 0.86 \| 1.12 \| \|  \| EMBARC \| -0.12 \| 0.06 \| 0.040 \| -0.24 \| -0.007 \|  \| 1.05 \| 0.803 \| 0.72 \| 1.53 \|  \| 1.09 \| 0.644 \| 0.74 \| 1.61 \| \|  \| **Meta-analysis** \| **-0.04** \| **0.03** \| **0.174** \| **-0.09** \| **0.02** \|  \| **1.00** \| **0.971** \| **0.86** \| **1.16** \|  \| **1.01** \| **0.832** \| **0.91** \| **1.12** \| \| Energy loss \| iSPOT \| 0.0004 \| 0.03 \| 0.999 \| -0.06 \| 0.06 \|  \| 1.04 \| 0.580 \| 0.91 \| 1.19 \|  \| 1.03 \| 0.683 \| 0.90 \| 1.17 \| \|  \| COMED \| 0.07 \| 0.03 \| 0.012* \| 0.02 \| 0.13 \|  \| 0.84 \| 0.046* \| 0.71 \| 1.00 \|  \| 0.72 \| 0.0001*** \| 0.61 \| 0.85 \| \|  \| GENDEP \| 0.07 \| 0.02 \| 0.003** \| 0.02 \| 0.117 \|  \| 0.88 \| 0.081 \| 0.76 \| 1.02 \|  \| 0.84 \| 0.017* \| 0.73 \| 0.97 \| \|  \| EMBARC \| -0.02 \| 0.06 \| 0.793 \| -0.14 \| 0.11 \|  \| 1.00 \| 0.998 \| 0.7 \| 1.43 \|  \| 1.00 \| 0.982 \| 0.7 \| 1.45 \| \|  \| **Meta-analysis** \| **0.04** \| **0.02** \| **0.044*** \| **0.001** \| **0.08** \|  \| **0.93** \| **0.187** \| **0.83** \| **1.04** \|  \| **0.87** \| **0.136** \| **0.73** \| **1.04** \| \| Leaden Paralysis \| COMED \| 0.07 \| 0.03 \| 0.012* \| 0.02 \| 0.12 \|  \| 0.88 \| 0.131 \| 0.75 \| 1.04 \|  \| 0.88 \| 0.108 \| 0.75 \| 1.03 \| \|  \| GENDEP \| 0.05 \| 0.02 \| 0.048* \| 0.004 \| 0.096 \|  \| 0.82 \| 0.007** \| 0.71 \| 0.95 \|  \| 0.73 \| 0.00004*** \| 0.63 \| 0.85 \| \|  \| **Meta-analysis** \| **0.05** \| **0.02** \| **0.001**** \| **0.02** \| **0.09** \|  \| **0.85** \| **0.001**** \| **0.76** \| **0.94** \|  \| **0.80** \| **0.016*** \| **0.66** \| **0.96** \| \| AES severity \| **Meta-analysis** \| **0.02** \| **0.02** \| **0.222** \| **-0.01** \| **0.06** \|  \| **0.91** \| **0.050** \| **0.82** \| **1.00** \|  \| **0.89** \| **0.089** \| **0.78** \| **1.02** \| |
| --- | --- | --- | --- | --- | --- | --- | --- | --- | --- | --- | --- | --- | --- | --- | --- | --- | --- | --- | --- | --- | --- | --- | --- | --- | --- | --- | --- | --- | --- | --- | --- | --- | --- | --- | --- | --- | --- | --- | --- | --- | --- | --- | --- | --- | --- | --- | --- | --- | --- | --- | --- | --- | --- | --- | --- | --- | --- | --- | --- | --- | --- | --- | --- | --- | --- | --- | --- | --- | --- | --- | --- | --- | --- | --- | --- | --- | --- | --- | --- | --- | --- | --- | --- | --- | --- | --- | --- | --- | --- | --- | --- | --- | --- | --- | --- | --- | --- | --- | --- | --- | --- | --- | --- | --- | --- | --- | --- | --- | --- | --- | --- | --- | --- | --- | --- | --- | --- | --- | --- | --- | --- | --- | --- | --- | --- | --- | --- | --- | --- | --- | --- | --- | --- | --- | --- | --- | --- | --- | --- | --- | --- | --- | --- | --- | --- | --- | --- | --- | --- | --- | --- | --- | --- | --- | --- | --- | --- | --- | --- | --- | --- | --- | --- | --- | --- | --- | --- | --- | --- | --- | --- | --- | --- | --- | --- | --- | --- | --- | --- | --- | --- | --- | --- | --- | --- | --- | --- | --- | --- | --- | --- | --- | --- | --- | --- | --- | --- | --- | --- | --- | --- | --- | --- | --- | --- | --- | --- | --- | --- | --- | --- | --- | --- | --- | --- | --- | --- | --- | --- | --- | --- | --- | --- | --- | --- | --- | --- | --- | --- | --- | --- | --- | --- | --- | --- | --- | --- | --- | --- | --- | --- | --- | --- | --- | --- | --- | --- | --- | --- | --- | --- | --- | --- | --- | --- | --- | --- | --- | --- | --- | --- | --- | --- | --- | --- | --- | --- | --- | --- | --- | --- | --- | --- | --- | --- | --- | --- | --- | --- | --- | --- | --- | --- | --- | --- | --- | --- | --- | --- | --- | --- | --- | --- | --- | --- | --- | --- | --- | --- | --- | --- | --- | --- | --- | --- | --- | --- | --- | --- | --- | --- | --- | --- | --- | --- | --- | --- | --- | --- | --- | --- | --- | --- | --- | --- | --- | --- | --- | --- | --- | --- | --- | --- | --- | --- | --- | --- | --- | --- | --- | --- | --- | --- | --- | --- | --- | --- | --- | --- | --- | --- | --- | --- | --- | --- | --- | --- | --- | --- | --- | --- | --- | --- | --- | --- | --- | --- | --- | --- | --- | --- | --- | --- | --- | --- | --- | --- | --- | --- | --- | --- | --- | --- | --- | --- | --- | --- | --- | --- | --- | --- | --- | --- | --- | --- | --- | --- | --- | --- | --- | --- | --- | --- | --- | --- | --- | --- | --- | --- | --- | --- | --- | --- | --- | --- | --- | --- | --- | --- | --- | --- | --- | --- | --- | --- |
|  |

Note. Linear mixed model analyses were conducted with individual atypical, energy-related symptoms as the independent variable and depressive symptoms severity during follow-up as dependent variable, adjusted for age, gender, baseline depression severity and time (linear and quadratic) within the individual studies and then pooled using random-effects meta-analyses. In addition, logistic regression analyses with individual atypical, energy-related symptoms as the independent variable and response and remission as dependent variable were conducted, adjusted for age and sex within the individual studies and then pooled using random-effects meta-analyses.

Supplementary Table 4: Categorical definitions of obesity and inflammation as predictors of treatment outcomes.

Table 4.1 Results from the meta analyses of associations between categorical obesity (BMI > 30 kg/m^2^) and inflammation and depression severity (adjusted for age, sex, linear and quadratic time and baseline depression severity).

|  |  | **Response** | | | |
| --- | --- | --- | --- | --- | --- |
| **Predictor** | **Study** | **OR** | **pvalue** | **95% CI** | |
| Obesity | COMED | 1.14 | 0.426 | 0.83 | 1.56 |
|  | iSPOT | 1.26 | 0.126 | 0.94 | 1.69 |
|  | GENDEP | 0.82 | 0.338 | 0.54 | 1.24 |
|  | EMBARC | 0.35 | 0.034* | 0.14 | 0.92 |
|  | **Meta-analysis** | **0.96** | **0.823** | **0.69** | **1.13** |
|  | Heterogeneity | Q(3) = 8.14, p = 0.043, I2 = 64.62% | | | |
| CRP >1 | COMED | 0.64 | 0.197 | 0.32 | 1.27 |
|  | GENDEP | 1.05 | 0.866 | 0.62 | 1.78 |
|  | EMBARC | 0.86 | 0.741 | 0.36 | 2.08 |
|  | **Meta-analysis** | **0.87** | **0.457** | **0.59** | **1.26** |
|  | Heterogeneity | Q(2) = 1.21, p = 0.547, I2 = 0% | | | |
| CRP > 3 | COMED | 0.63 | 0.172 | 0.32 | 1.23 |
|  | GENDEP | 1.57 | 0.192 | 0.79 | 3.11 |
|  | EMBARC | 1.02 | 0.965 | 0.36 | 2.88 |
|  | **Meta-analysis** | **1.00** | **0.987** | **0.55** | **1.80** |
|  | Heterogeneity | Q(2) = 3.51, p = 0.173, I2 = 44.88% | | | |

Table 4.2 Results from the meta analyses of associations between categorical obesity (BMI > 30 kg/m^2^) and inflammation and treatment response (adjusted for age and sex).

|  |  | **Depressive symptom severity** | | | | |
| --- | --- | --- | --- | --- | --- | --- |
| **Predictor** | **Study** | **β** | **SE** | **pvalue** | **95% CI** | |
| Obesity | COMED | -0.07 | 0.05 | 0.172 | 0.85 | 1.03 |
|  | iSPOT | -0.02 | 0.05 | 0.752 | 0.89 | 1.08 |
|  | GENDEP | 0.18 | 0.07 | 0.008** | 1.04 | 1.37 |
|  | EMBARC | 0.02 | 0.15 | 0.884 | 0.76 | 1.37 |
|  | **Meta-analysis** | **0.02** | **0.06** | **0.728** | **-0.09** | **0.13** |
|  | Heterogeneity | Q(3) = 8.75, p = 0.033, I2 = 68.57% | | | | |
| CRP >1 | COMED | 0.14 | 0.12 | 0.242 | 0.91 | 1.46 |
|  | GENDEP | 0.13 | 0.09 | 0.149 | 0.95 | 1.36 |
|  | EMBARC | 0.13 | 0.15 | 0.387 | 0.85 | 1.53 |
|  | **Meta-analysis** | **0.13** | **0.06** | **0.041*** | **0.01** | **0.26** |
|  | Heterogeneity | Q(2) = 0.01, p= 0.998, I2 = 0% | | | | |
| CRP > 3 | COMED | 0.13 | 0.13 | 0.265 | 0.92 | 1.41 |
|  | GENDEP | 0.15 | 0.11 | 0.165 | 0.94 | 1.44 |
|  | EMBARC | 0.07 | 0.17 | 0.684 | 0.77 | 1.50 |
|  | **Meta-analysis** | **0.13** | **0.07** | **0.090** | **-0.02** | **0.28** |
|  | Heterogeneity | Q(2) = 0.16, p-val = 0.925, I2 = 0% | | | | |

|  |  | **Remission** | | | |
| --- | --- | --- | --- | --- | --- |
| **Predictor** | **Study** | **OR** | **pvalue** | **95% CI** | |
| Obeisity | COMED | 1.18 | 0.296 | 0.87 | 1.62 |
|  | iSPOT | 1.35 | 0.044* | 1.01 | 1.81 |
|  | GENDEP | 0.62 | 0.041* | 0.40 | 0.98 |
|  | EMBARC | 0.52 | 0.173 | 0.20 | 1.32 |
|  | **Meta-analysis** | **0.95** | **0.781** | **0.63** | **1.41** |
|  | Heterogeneity | Q(3) = 10.66, p = 0.015, I2 = 75.72% | | | |
| CRP >1 | COMED | 0.98 | 0.959 | 0.50 | 1.91 |
|  | GENDEP | 0.57 | 0.050 | 0.32 | 1.00 |
|  | EMBARC | 0.78 | 0.581 | 0.32 | 1.92 |
|  | **Meta-analysis** | **0.73** | **0.110** | **0.49** | **1.07** |
|  | Heterogeneity | Q(2) = 1.50, p = 0.473, I2= 0% | | | |
| CRP > 3 | COMED | 0.86 | 0.646 | 0.45 | 1.64 |
|  | GENDEP | 1.42 | 0.317 | 0.71 | 2.82 |
|  | EMBARC | 1.63 | 0.366 | 0.57 | 4.7 |
|  | **Meta-analysis** | **1.16** | **0.489** | **0.76** | **1.79** |
|  | Heterogeneity | Q(2) = 1.55, p = 0.460, I2 = 0% | | | |

Table 4.3 Results from the meta analyses of associations between categorical obesity (BMI > 30 kg/m^2^) and inflammation and remission (adjusted for age and sex)

Supplementary Table 5: Meta-analyses on SSRI arms only.

|  |  | **Depressive symptom severity** | | | | | |
| --- | --- | --- | --- | --- | --- | --- | --- |
| **Predictor** | **Study** | **n** | **β** | **SE** | **pvalue** | **95% CI** | |
| AES Severity | COMED | 213 | 0.004 | 0.05 | 0.927 | -0.09 | 0.10 |
|  | iSPOT | 646 | -0.01 | 0.03 | 0.703 | -0.07 | 0.05 |
|  | GENDEP | 420 | 0.07 | 0.03 | 0.026* | 0.01 | 0.13 |
|  | EMBARC | 138 | -0.06 | 0.06 | 0.320 | -0.18 | 0.06 |
|  | **Meta-analysis** | **1417** | **0.01** | **0.02** | **0.697** | **-0.04** | **0.06** |
|  | Heterogeneity | Q(3) = 5.77, p = 0.123, I2 = 48.01% | | | | | |
| BMI | COMED | 213 | 0.05 | 0.05 | 0.316 | -0.05 | 0.14 |
|  | iSPOT | 635 | -0.01 | 0.03 | 0.775 | -0.07 | 0.05 |
|  | GENDEP | 429 | 0.03 | 0.03 | 0.323 | -0.03 | 0.09 |
|  | EMBARC | 128 | 0.05 | 0.07 | 0.448 | -0.09 | 0.19 |
|  | **Meta-analysis** | **1405** | **0.02** | **0.02** | **0.239** | **-0.01** | **0.06** |
|  | Heterogeneity | Q(3) = 1.60, p = 0.660, I2 = 0% | | | | | |
| CRP | COMED | 47 | 0.15 | 0.10 | 0.135 | -0.04 | 0.43 |
|  | GENDEP | 106 | 0.21 | 0.06 | 8.31e-04*** | 0.09 | 0.33 |
|  | EMBARC | 100 | 0.08 | 0.08 | 0.331 | -0.08 | 0.24 |
|  | **Meta-analysis** | **253** | **0.16** | **0.04** | **0.0002***** | **0.08** | **0.24** |
|  | Heterogeneity | Q(2) = 1.65, p = 0.439, I2 = 1.93% | | | | | |
| IMD index | COMED | 47 | 0.20 | 0.12 | 0.109 | -0.04 | 0.43 |
|  | GENDEP | 103 | 0.39 | 0.09 | 6.75e-04*** | 0.21 | 0.56 |
|  | EMBARC | 92 | 0.01 | 0.11 | 0.910 | -0.21 | 0.23 |
|  | **Meta-analysis** | **242** | **0.20** | **0.12** | **0.079** | **-0.02** | **0.43** |
|  | Heterogeneity | Q(2) = 7.05, p = 0.029, I2 = 70.24% | | | | | |

Supplementary Table 6: Meta-analyses in males and females separately.

Table 6.1 Associations between baseline IMD features and follow-up depressive symptom severity in males only.

| **Predictor** | **Study** | **n** | **β** | **SE** | **pvalue** | **95% CI** | |
| --- | --- | --- | --- | --- | --- | --- | --- |
| AES Severity | COMED | 202 | 0.01 | 0.05 | 0.851 | -0.09 | 0.11 |
|  | iSPOT | 419 | 0.04 | 0.04 | 0.312 | -0.04 | 0.12 |
|  | GENDEP | 270 | 0.10 | 0.04 | 0.011* | 0.02 | 0.18 |
|  | EMBARC | 42 | 0.07 | 0.11 | 0.520 | -0.15 | 0.29 |
|  | **Meta-analysis** | **933** | **0.06** | **0.02** | **0.019*** | **0.01** | **0.10** |
| BMI | COMED | 200 | -0.07 | 0.05 | 0.156 | -0.17 | 0.03 |
|  | iSPOT | 413 | 0.02 | 0.04 | 0.634 | -0.06 | 0.10 |
|  | GENDEP | 279 | 0.08 | 0.04 | 0.034* | 0.002 | 0.16 |
|  | EMBARC | 39 | 0.001 | 0.13 | 0.992 | -0.25 | 0.25 |
|  | **Meta-analysis** | **931** | **0.01** | **0.04** | **0.682** | **-0.05** | **0.08** |
| CRP | COMED | 45 | 0.16 | 0.12 | 0.168 | -0.08 | 0.40 |
|  | GENDEP | 80 | 0.11 | 0.07 | 0.106 | -0.03 | 0.25 |
|  | EMBARC | 32 | -0.11 | 0.13 | 0.431 | -0.36 | 0.14 |
|  | **Meta-analysis** | **157** | **0.07** | **0.07** | **0.286** | **-0.06** | **0.21** |
| IMD index | COMED | 45 | -0.04 | 0.16 | 0.829 | -0.35 | 0.27 |
|  | GENDEP | 77 | 0.23 | 0.11 | 0.036* | 0.01 | 0.45 |
|  | EMBARC | 29 | -0.14 | 0.23 | 0.553 | -0.59 | 0.31 |
|  | **Meta-analysis** | **151** | **0.07** | **0.12** | **0.545** | **-0.16** | **0.30** |

| **Predictor** | **Study** | **n** | **β** | **SE** | **pvalue** | **95% CI** | |
| --- | --- | --- | --- | --- | --- | --- | --- |
| AES Severity | COMED | 431 | 0.02 | 0.03 | 0.622 | -0.04 | 0.07 |
|  | iSPOT | 548 | -0.003 | 0.03 | 0.931 | -0.06 | 0.06 |
|  | GENDEP | 475 | 0.02 | 0.03 | 0.525 | -0.04 | 0.08 |
|  | EMBARC | 96 | -0.15 | 0.07 | 0.028* | -0.29 | -0.01 |
|  | **Meta-analysis** | **1550** | **-0.003** | **0.02** | **0.888** | **-0.05** | **0.04** |
| BMI | COMED | 430 | 0.01 | 0.03 | 0.837 | -0.05 | 0.06 |
|  | iSPOT | 543 | -0.03 | 0.03 | 0.419 | -0.08 | 0.03 |
|  | GENDEP | 483 | 0.04 | 0.03 | 0.206 | -0.02 | 0.10 |
|  | EMBARC | 89 | 0.05 | 0.08 | 0.512 | -0.11 | 0.21 |
|  | **Meta-analysis** | **1545** | **0.01** | **0.02** | **0.612** | **-0.02** | **0.04** |
| CRP | COMED | 112 | 0.001 | 0.06 | 0.991 | -0.12 | 0.12 |
|  | GENDEP | 139 | 0.04 | 0.06 | 0.492 | -0.08 | 0.16 |
|  | EMBARC | 68 | 0.13 | 0.08 | 0.138 | -0.03 | 0.29 |
|  | **Meta-analysis** | **319** | **0.03** | **0.03** | **0.375** | **-0.03** | **0.09** |
| IMD index | COMED | 112 | 0.13 | 0.09 | 0.126 | -0.04 | 0.31 |
|  | GENDEP | 138 | 0.19 | 0.08 | 0.024* | 0.03 | 0.35 |
|  | EMBARC | 63 | -0.14 | 0.23 | 0.553 | -0.59 | 0.31 |
|  | **Meta-analysis** | **313** | **0.14** | **0.06** | **0.013*** | **0.03** | **0.26** |

Table 6.2 Associations between baseline IMD features and follow-up depressive symptom severity in females only.
